# Why Cancer & Metabolism? Why now?

**DOI:** 10.1186/2049-3002-1-1

**Published:** 2013-01-23

**Authors:** Chi Van Dang, Michael Pollak

**Affiliations:** 1 Abramson Cancer Center, 3400 Spruce Street, Philadelphia, PA 19104, USA; 2 2 Segal Cancer Centre and McGill University, 3755 Cote-Ste-Catherine, Montreal, Quebec H3T 1E2, Canada

## 

The identification of oncogenic driver mutations of many cancers by deep sequencing is validating the oncogene versus tumor suppressor paradigm of tumorigenesis. At the same time, there is a profound resurgence of interest in metabolism in the context of neoplasia, both at the whole-organism level with respect to the influence of caloric intake on cancer behavior, and at the cellular level, with respect to possible therapeutic exploitation of differences between the metabolism of normal and cancer cells. The rapid expansion of research on metabolic aspects of neoplasia has improved our understanding of how oncogenes and tumor suppressors are linked to altered cancer cell metabolism, and how altered metabolism, in turn, affect the cancer epigenome. Enzymes of key metabolic pathways are mutated in specific types of cancers, linking oncogenic alterations to perturbed metabolism. Newly identified metabolic pathways have also emerged as the flexibility of re-wired cancer cell metabolism is appreciated. Much of this development has been enabled by better tools to study the genome as well as cellular metabolism.

At the organismal level, the clinical association of obesity with increased cancer risk, the classic observations that caloric restriction can inhibit carcinogenesis in rodent models, and experimental models that suggest that the behavior of a subset of cancers is influenced by drugs such as metformin, that may act at least in part by perturbing whole organism energy metabolism, further tie altered metabolic states with tumorigenesis and cancer progression. Improvements in metabolic imaging have also provided new glimpses of in vivo real-time metabolic changes. Both hyperpolarized 13C MRI and new positron emission tomography (PET) radiolabeled ligands provide remarkable insights into tumor metabolism in vivo. The journal *Cancer* &*Metabolism* provides a timely forum to report progress in cancer research spanning the entire spectrum including cell metabolism, metabolic imaging, whole organism metabolism, circadian influences on metabolism, and clinical studies.

Why *Cancer* &*Metabolism* now? It is a journal overseen by practicing scientists for scientists, to offer a rapid means to communicate research findings in this booming field. The journal aims for rapid turn around and minimal revisions limited only to those that may be required to substantively support the major conclusions drawn in the title and abstract of the paper. The journal will also provide timely critical reviews in areas of this rapidly changing field. All articles will be published online and open access soon after acceptance, thus providing a rapidly growing forum for significant research. A brief history of the field below will underscore what has brought us to this point, as well as our predictions for this exciting, turbulent area of research.

The discoveries of major metabolic pathways decades ago by notables, such as Krebs, Warburg, Embden, Myerhof, Kennedy, and others, laid the foundation for the use of experimental methods to study the metabolism of cancer. Otto Warburg has remained to date as the most prominent contributor to our early understanding of cancer metabolism, with aerobic glycolysis or the Warburg effect recognized as a hallmark of cancer. This apparent reversion of cancer cells to a primitive form of energetic metabolism, as compared to oxidative phosphorylation, was thought to contribute directly to the development of cancer well before the identification of oncogenes and tumor suppressors. While recent data require some refinement of Warburg’s conclusions, the impact of his pioneering work remains considerable. It is instructive to recognize that Warburg detected metabolic features of neoplastic cells as distinct from untransformed cells with relatively simple methods, well before the complexities of oncogenic signaling networks had been recognized.

The discovery of cancer genes took the center stage of cancer research for several decades, providing significant insight into the development of cancer. However, beyond the molecular switches controlled by genes that turn growth and proliferative programs on and off, little was known about how a growing cancer cell coordinates growth signaling with nutrient uptake for an orderly and balanced assembly of new cellular components of the growing cell. In retrospect, it is unsurprising that many cancer genes are directly connected with the regulation of cell metabolism in order that adequate amounts of ATP, carbon skeletons and nitrogen are acquired and channeled into macromolecular synthesis. Importantly, while Warburg focused on glucose metabolism, we now know that the cancer cell utilizes a variety of nutrient sources, not only by transport of raw nutrients such as glucose and glutamine into the cells, but also by resorting to autophagy and macropinocytosis to eat themselves or the surrounding nutrient-rich circulating proteins and lipids.

As with any rapidly emerging field, we anticipate the leading edge of findings to be turbulent, provocative, and controversial, only to settle in the calmer wake of established facts that endure the test of time. Hence, *Cancer* &*Metabolism* expects to publish provocative and controversial findings as long as the scientific merit of the work holds up to fair peer review.

Peeking into the future, we expect that hypotheses concerning the efficacy of “metabolic therapies”, such as the use of biguanides (to inhibit mitochondrial Complex I and activate AMPK) or chloroquine (to inhibit autophagy), and others will be tested in the clinic, and that results will stimulate new lines of investigation that will build on early hypotheses. Perhaps mechanisms linking obesity to cancer risk will rest on new activities of adipokines whose altered levels could directly affect cancer cells. Insights into how the circadian regulation of metabolism could affect tumorigenesis or could be exploited for therapy may be forthcoming. Although caloric restriction as it relates to longevity may be controversial, as the result of a recent study in monkeys shows, its role in cancer development may be further revealed not only through genetically engineered mouse models of cancers, but also through more sophisticated population studies where metabolic characteristics are quantified. The role of mitophagy (the removal of mitochondria via the autophagic machinery, particularly during nutrient deprivation) may prove to be important to cancer development, and involve processes such as increased oxidative stress attributable to failed mitophagy.

Hypoxia, which is prevalent in cancers, may be exploited for therapeutic purposes through direct effective targeting of HIF or its targets. Drug candidates that target specific enzymes, such as fatty acid synthase, glutaminase, lactate dehydrogenase, pyruvate dehydrogenase kinase 1, pyruvate kinase, or those targeting metabolic transporters, such as MCT1 and GLUT1, may appear in the next few years from the efforts of many companies and academic laboratories. Evaluation of these agents may require companion diagnostics, and may offer important opportunities for synthetic lethality in combination with other drugs. The complexity of the tumor microenvironment will reveal not only cell intrinsic tumor heterogeneity but also the complex features of obligate dependency of metabolic programs of stromal cells on those of the cancer cells and vice versa.

The rapid pace of discovery in this field will benefit from an open access forum such as *Cancer* &*Metabolism* to disseminate information and to promote an area of investigation that holds promise for both curiosity driven basic research and clinically directed research as we seek to improve the diagnosis and therapy of cancer. The time is now, and the place to publish a wide spectrum of work on cancer metabolism is *Cancer* &*Metabolism*. We look forward to many exciting volumes after this inaugural issue, which provides examples of the promising research and excitement in the field.

